# Decoding the role of oxidative stress resistance and alternative carbon substrate assimilation in the mature biofilm growth mode of *Candida glabrata*

**DOI:** 10.1186/s12866-024-03274-9

**Published:** 2024-04-20

**Authors:** Khem Raj, Dhiraj Paul, Praveen Rishi, Geeta Shukla, Dhiraj Dhotre, YogeshSouche

**Affiliations:** 1 Department of Microbiology Basic Medical Sciences Block I, South Campus, Panjab University, Sector-25, Chandigarh, 160014 India; 2 Department of Environmental and Biological Sciences, University of Eastern Finland, Kuopio, Finland; 3 National Centre for Microbial Resource, National Centre for Cell Sciences (NCCS), Pune, India

**Keywords:** *C. glabrata*, Biofilm, Transcriptomics, Oxidative stress, Alternative carbon substrate assimilation

## Abstract

**Background:**

Biofilm formation is viewed as a vital mechanism in *C. glabrata* pathogenesis. Although, it plays a significant role in virulence but transcriptomic architecture and metabolic pathways governing the biofilm growth mode of *C. glabrata* remain elusive. The present study intended to investigate the genes implicated in biofilm growth phase of *C. glabrata* through global transcriptomic approach.

**Results:**

Functional analysis of Differentially expressed genes (DEGs) using gene ontology and pathways analysis revealed that upregulated genes are involved in the glyoxylate cycle, carbon-carbon lyase activity, pre-autophagosomal structure membrane and vacuolar parts whereas, down- regulated genes appear to be associated with glycolysis, ribonucleoside biosynthetic process, ribosomal and translation process in the biofilm growth condition. The RNA-Seq expression of eight selected DEGs (Cg*ICL1*, Cg*MLS1*, Cg*PEP1*, and Cg*NTH1*, Cg*ERG9*, Cg*ERG11*, Cg*TEF3*, and Cg*COF1*) was performed with quantitative real-time PCR (RT-qPCR). The gene expression profile of selected DEGs with RT-qPCR displayed a similar pattern of expression as observed in RNA-Seq. Phenotype screening of mutant strains generated for genes Cg*PCK1* and Cg*PEP1*, showed that Cg*pck1*∆ failed to grow on alternative carbon substrate (Glycerol, Ethanol, Oleic acid) and similarly, Cg*pep1*∆ unable to grow on YPD medium supplemented with hydrogen peroxide. Our results suggest that in the absence of glucose, *C. glabrata* assimilate glycerol, oleic acid and generate acetyl coenzyme-A (acetyl-CoA) which is a central and connecting metabolite between catabolic and anabolic pathways (glyoxylate and gluconeogenesis) to produce glucose and fulfil energy requirements.

**Conclusions:**

The study was executed using various approaches (transcriptomics, functional genomics and gene deletion) and it revealed that metabolic plasticity of *C. glabrata* (NCCPF-100,037) in biofilm stage modulates its virulence and survival ability to counter the stress and may promote its transition from commensal to opportunistic pathogen. The observations deduced from the present study along with future work on characterization of the proteins involved in this intricate process may prove to be beneficial for designing novel antifungal strategies.

**Supplementary Information:**

The online version contains supplementary material available at 10.1186/s12866-024-03274-9.

## Introduction

Candidemia, and other forms of invasive candidiasis (IC) caused by different *Candida* species are unquestionably the most prevalent form of invasive mycoses worldwide, especially in paediatric wards and intensive care units (ICUs) [1–3].Individuals suffering with diabetes, immune deficiencies, hematologic malignancies as well those using indwelling medical devices, receiving parenteral nutrition or undergoing prior surgeries due to other morbidities are most susceptible to candidemia and IC [4–6]. A global antifungal surveillance program, reported that patients suffering from candidemia and IC had a remarkable shift in the prevalence of Candida species towards non-albicans Candida (NAC) species [3]. Amongst NAC species, *Candida glabrata* has been reported to be the leading pathogen accounting for 10–35% of total bloodstream infections that alone causes 30–90% mortality [3, 7, 8]. Moreover, the elevated emerging incidence of *C. glabrata* infections have raised serious health concerns in the recent past due to its inherent drug resistance towards azole-based antifungal drugs, co-resistance to the amphotericin-B and echinocandins (the latest antifungal agent) as well as stealth immune evasion strategy, making it difficult to manage and treat in ICU settings [2, 3, 9]. *C. glabrata*, a haploid yeast, is mainly found as a commensal in the oral cavity, gut, and vaginal tract of humans but in immunocompromised individuals it may cause candidemia and candidiasis due to its virulence attributes such as phospholipases, lipases, haemolysins, GPI-linked aspartyl proteases and having intracellular replicative capacity within host macrophages [10, 11]. More specifically, *C. glabrata* has a strong biofilm formation potential making it more resistant to various interventions [9, 12, 13]. Biofilm formation is a potent virulence factor for the pathogenesis of *C. glabrata* as it forms protective sheath that imparts antifungal drug resistance by mainly inhibiting the penetrating ability of antifungal drugs vis-a-vis eradication of biofilm leading to persistence and dissemination of infection in host and helps in immune evasion of the host to provide an impeccable strategy in clinical infection [14, 15]. Biofilm resistance in Candida species has been reported because of overexpression of efflux pumps, composition cell wall, increased cell density and presence of thick extracellular matrix [16–18]. The environmental stress conditions, nutritional access, and availability of oxygen determine the biological activities of cells in biofilm growth mode [19]. In this context, a few key questions need be addressed to understand the pathogenomics of biofilm of the *C. glabrata* such as (i) What are the systemic level alterations occurring in the mature biofilm cells of *C. glabrata* and (ii) How differential expression of gene regulate the cellular and molecular functions that contribute to the stress resistance in the biofilm. Thus, it is pertinent to identify the genes and gene sets involved in the biological pathways and understand how they maintain the integrity of mature biofilm and disseminate *C. glabrata* infection in the host. Moreover, various alterations occur in the biofilm cells at genomics, transcriptomics and proteomics level. Although, few candidate genes have been observed to be associated with biofilm formation in *C. glabrata* [12, 20, 21], but detailed investigations of how large gene sets and their regulatory networks shape this intricate process has not been deciphered yet. Transcriptomic studies have well documented the profiling of differential expression of genes (DEGs) in varied environmental and stress conditions such as pH, nitrosative, and antifungal stress encountered by *C. glabrata* [22–24].However, the differential expression of genes and how their functional attributes regulate the metabolic path-ways and biological processes to withstand the varied stresses (environmental stress, antifungal stress and host immune response) prevailing in the mature biofilm of *C. glabrata* still needed to be elucidated. Therefore, comparative transcriptome profiling between planktonic and biofilm conditions in *C. glabrata* (NCPPF-100,037) along with the functional characterisation of the significantly upregulated gene, may provide useful information about the regulatory network of genes and its pathotranscriptomics. Gene set enrichment analysis, metabolic pathways, and functional analysis of DEGs associated with the biofilm growth phase of *C. glabrata*, facilitate the identification and functional visualization of the genes implicated in metabolic network re-wiring in response to the stress conditions prevailing in this phase and augmented its virulence fitness attributes. Thus, for a better understanding of pathobiology in *C. glabrata* at the transcriptomic and molecular level, the present study was designed with an aim to decipher differentially expressed genes (DEGs), vis-a-vis functional characterization of selected genes involved in the biofilm formation of *C. glabrata* (NCCPF-100,037).

## Methods

All the clinical strains of *C. glabrata* (NCCPF-100,029, NCCPF-100,033, NCCPF- 100,037) and standard strain (ATCC-2001) were procured from National Culture Collection of Pathogenic Fungi (NCCPF), Post Graduate Institute of Medical and Education Research (PGIMER), Chandigarh, India. The standard strain of *C. glabrata* (MTCC-3019) was obtained from the Institute of Microbial Technology (IMTECH), Chandigarh, India.

### Quantification of biofilm forming potential

i. **Crystal violet assay**: 100 µL of log phase culture (10^6^ CFU/mL) of all *C. glabrata* strains were added to polystyrene 96-well microtiter plate in SDB medium and medium without any cells served as a control. The plates were incubated for 24 h at 37 °C and thereafter, medium was carefully aspirated without disrupting the biofilm formed, washed with sterile PBS followed by addition of 0.2% crystal violet and incubated at 37 °C for 10–15 min. Plates were washed with PBS, dried, 100 µL of 30% acetic acid was added to each well to solubilize the stain and again incubated at 37 °C for 10–15 min. Solubilized crystal violet (100 µL) was transferred to a fresh microtiter plate and optical density (O.D) was measured at 550 nm using a microtiter plate reader (Biotek Microplate reader, USA) with 30% acetic acid as a blank [25, 26].
ii. **XTT assay (2,3-Bis-(2-Methoxy-4-Nitro-5-Sulfophenyl)-2 H-Tetrazolium-5- Carboxanilide)** :100 µL of XTT/menadione solution was added to each well containing a pre-washed biofilm as well as to the negative controls. The plates were incubated in the dark for 2 h at 37 °C. The supernatant (80 µL) from each well was transferred to a new microtiter plate and the absorbance was measured at 490 nm using a microtiter plate reader (Biotek Microplate reader, USA) [25].
iii. **Confocal laser scanning microscopy (CLSM)** :Biofilm was prepared over coverslip in a 6-well tissue culture plate. The conditions for biofilm formation were the same as described in Sect. 2(i). Once the biofilm is formed over the coverslips, 2 mL of dye mix was added containing FUN-1[2 chloro 4(2,3 dihydro-3-methyl (benzo-1,3-thiazol 2-yl)-methylidene) 1 phenyl quinolinium iodide] in PBS and incubated for 30–35 min at 37 °C. The coverslip was then gently removed from the dye mix and placed on a 35 mm glass bottom petri dish. Biofilm was examined under confocal scanning laser microscope at 63.3X (Leica SP5 II system, Leica Microsystems, India) [27]. Z-stack images were also captured at the same magnification for 3-D image reconstruction.

### RNA sequencing

Clinical isolate *C. glabrata* (NCCPF-100,037), showing the best biofilm forming ability was selected for RNA isolation and sequencing. Briefly, 100 µL of log phase culture (10^6^CFU/mL) was added in each well of the 6-well tissue culture plate and incubated at 37 °C for 24 h under static conditions. The biofilm cells from were scrapped off with a scrapper. Cells from biofilm and planktonic growth phases were subjected to enzymatic treatment with lyticase at 37 °C for 1 h. RNA was extracted using Hi-PurA yeast RNA purification kit (Hi-Media, Laboratories Pvt. Ltd. India) as per manufacturer’s instructions [28]. The concentration, purity and quality of isolated RNA was assessed using Nanodrop1000 spectrophotometer (Thermo Scientific, USA). Briefly, 1 µL of extracted RNA was used in the apparatus and RNA absorbance ratio was observed at 260 nm and 280 nm. The integrity of RNA was analysed by running the isolated RNA sample on 1.5% agarose gel. RNA sequencing of a total of six RNA samples (3 biological replicate from each biofilm and planktonic growth conditions) were outsourced from Agri-Genome Pvt. Ltd. Kochi, India. The RNA-Seq library was prepared following the Illumina TrueSeq protocol and RNA sequencing was performed with 250 bp long, paired end chemistry using the next generation sequencing (NGS) platform, Illumina HiSeq2500.

### RNA seq data analysis

i. **Quality check of raw reads** :The quality check of all raw reads was performed with FastQC tool. Parameters such as base quality score distribution, sequence quality score distribution, average base con- tent per read, GC distribution in the reads, PCR amplification issue, check for over-represented sequences, check for biasing of k-mers, and read-length distribution were analysed. Bad quality sequences were trimmed by the Trimmomatic tool and retained only high-quality sequence for further analysis. The whole RNA-Seq analysis was carried out with a command line script in Linux operating system. RNA-Seq raw reads generated in the study were submitted to the NCBI-GEO database under the bioproject with accession number PRJNA680327; GEO: GSE162024.
ii. **Analysis of differential expressed genes (DEGs)** :RNA-Seq analysis was performed after the quality check of each raw read using Tuxedo-2 pipeline, which comprises integrated bioinformatics tools (Tophat2, Cufflink, Cuffmerge, Cuffdiff). Processed clean reads of all samples from both the conditions (planktonic and biofilm) were aligned and mapped over the reference genome of *C. glabrata*, strain CBS138 downloaded from the Ensemble database (*C. glabrata*.ASM254v2.34.gtf.gz) with Tophat-2 program (version2.1.1) using default parameters of Bowtie 2 aligner. Aligned output files (in .bam format) generated from Tophat-2 were assembled with Cufflink tool [29–31]. Condition specific differential expression of genes was estimated using Cuffdiff by quantifying of assembled fragments or FPKM (fragment per kilo base pair of exons per million reads) or fragments count. Visualization and representation of DEGs was performed using Cummerbund and ggplot-2 packages of R [30, 32].
iii. **Functional classification, gene ontology and pathway analysis of DEGs** :Gene Ontology (GO) functions and Kyoto Encyclopedia of Genes and Genomes (KEGG) pathways analysis of DEGs (top upregulated and downregulated genes) were performed using ClueGo v2.5.7/Cluepedia v1.5.7 plugin of Cytoscape v3.7.0. Genome database of *C. glabrata* 138 was downloaded from the ClueGO application and gene IDs of DEGs were uploaded or directly pasted into the search panel of ClueGO Application. Gene ontology of DEGs was assigned according to cell components, molecular function and biological process of genes involved by selecting the various options of ClueGO. Gene enrichment analysis of gene sets associated with a particular metabolic pathway analysis was performed by choosing the KEGG database option and the refinement steps were included by selecting the network specificity as medium with an adjusted *p*-value (*p* < 0.0500) and radial layout options. Furthermore, gene IDs were assigned to each network using the Cluepedia application [33–35].

### Verification of DEGs with RT-qPCR

RNA-Seq expression of selected DEGs was verified with real-time quantitative PCR (RT-qPCR). RT-qPCR was performed with RNA isolated from mature biofilm (test) and planktonic cells (control) of *C. glabrata*. The cDNA was synthesized from RNA with a reverse transcription verso kit (Thermo Fisher Scientific, USA). RT-qPCR was performed with StepOnePlus real-time machine (Applied Biosystem, USA) using SYBER green PCR master mix (Applied Biosystem, USA) according to the manufacturer’s instructions. Each reaction mixture was set up in triplicate of 20 µL volume with 20 ng of cDNA and run for 40 cycles (Thermo-cycler conditions, initial holding step at 95 °C for 10 min, followed by 40 cycles at 95 °C each for 15 s and 60 °C for 1 min annealing/extension). The gene expression was determined using C_T_ method and normalized with housekeeping gene Cg*RDN18-1(*18 S-rDNA) [36]. A list of primers used to amplify the target genes is given in (Supplementary Table S1). The following equation was used to calculate the fold change of gene expression between biofilm and planktonic growth phase.

### Functional validation of selected genes

i. **Yeast colony PCR** :Zymolyase (MP Biomedicals) treatment was performed to digest the cell wall and obtain spheroplasts. For this, a zymolyase cocktail or digestion cocktail [zymolyase (2 mg/mL) and sorbitol (1.2 M)] was prepared in PBS and 10 µL aliquots were dispensed into 0.2 mL PCR tubes. A tip-full of yeast cells from a single colony or purified yeast transformant were added to the tubes and incubated at 37 °C for 3–4 h. The PCR mix was made with the appropriate primer set and 1 µL of the zymolyase-digested cell suspension was added as a template to perform PCR. Gel extraction for the amplified PCR product was performed using QIAgen kits (QIAgen Pvt Ltd, Germany) and protocols were followed, according to the manufacturer’s instructions.
ii. **Cassettes construction** :Open reading frames (ORFs) of Cg*PCK1* andCg*PEP1* of *C. glabrata* were deleted by homologous recombination with *Nat1* gene deletion cassette [37]. A cassette containing the *Nat1* gene was used, which encodes nourseothricin acetyltransferase (NAT) and imparts resistance to nourseothricin and replaces the gene of interest with the *Nat1*, the sequence of pRK625 plasmid is presented in appendix-A. The *Nat1* gene was amplified using PCR from the pRK625 plasmid in two halves carrying a common 310 bp overlapping region between them. Subsequently, the 5’ and 3’ untranslated regions (UTR) of the target gene from genomic DNA from the wild-type clinical isolate of *C. glabrata* were amplified using respective primers with amplified PCR products of approximately 500–550 bp size. The *Nat1* deletion cassette was synthesized by fusion PCR from both 5’- and 3’- UTR region of a target gene (Cg*PCK1 and* Cg*PEP1*) and each half of the *Nat1* gene derived from amplification of pRK625 plasmid using overhang primers (fusion product 1 (FP1) = 5’ nat1 + 5’UTR region of a target gene, fusion product 2 (FP2) = 3’ *Nat1* + 3’ UTR region of target gene). Both amplified *Nat1* half of fusion products FP1 and FP2 share 310 bp complementary regions. Both fused PCR products were Co-transformed into a wild type *C. glabrata* (NCCPF- 100,037), transformants were spread plated on YPD and incubated at 30 °C for 12–16 h so that homologous recombination between nat1 deletion cassette and wild type genomic loci may occur. Replica plating of incubated transformants was carried out on YPD supplemented with 200 mg/mL nourseothricin and incubated for a further 24 h. Colonies resistant to nourseothricin were selected out, genomic DNA was isolated and gene disruption of Cg*PCK1* andCg*PEP1* were checked and confirmed by PCR. All set of primers used for mutants generation and gene deletion confirmation are given in (Supplementary Table S2).
iii. **Transformation** :Transformation of a clinical isolate of *C. glabrata* was carried out using the lithium acetate method [38, 39]. *C. glabrata* were grown to log phase in YPD broth, cold centrifuged, and pelleted cells washed with PBS. Cells were re-suspended in 50 µL of 100 mM of lithium acetate and divided into 50 µL of aliquots for each transformant. Each tube with 50 µL of aliquot was added with transformation mixture (36 µL lithium acetate (1 M), 240 µL of 50% Polyethylene glycol (PEG), 5 µL of heat-denatured of single-stranded Salmon sperm DNA, and 1 µg of transforming DNA per sample). After gentle vortexing, incubated for 45 min at 30 °C and 43 µL of dimethyl sulfoxide (DMSO) was added followed by heat shock at 42 °C for 15 min. Post heat shock treatment it was kept at 37 °C for 5 min, cells were pelleted down and the supernatant discarded. Cell pellets were re-suspended in 1 mL of sterile water/PBS and 20 µL of cell suspension was spread plated on the selection media and incubated at 30 °C for 48 h.
iv. **Phenotype screening of mutant strains** : Wild type standard strain (ATCC-2001), wild type clinical isolate of *C. glabrata* (NCCPF-100,037) and mutants generated from clinical isolate were inoculated in 96 well plate and incubated at 30 °C for 24 h. 10fold serial dilution of each overnight grown culture was spotted onto YPD, YNB with Glucose 2% and YNB plates containing different alternative carbon substrates such as Glycerol 3%, Ethanol 2% and Oleic acid 2%. In a second set of plates, YNB were supplemented with metal stress inducers such as Zinc chloride (ZnCl_2_)-8 mM, Manganese chloride (MnCl)-3 mM, cell-wall stressor for yeast cell (Calcofluor white − 25 µg/mL, Menadione - and Fluconazole- 16 g/mL). The growth phenotype of mutant strains was analysed after incubation of 48–72 h at 30 °C.

### Statistical analysis

The GraphPad Prism software was used to plot data and graphical analysis. The two- tailed student’s t-test was used for comparative intergroup analysis and spearman’s correlation regression analysis was used for scatter plot of gene expression. A *p*-value of < 0.05 and with 5% FDR was considered significant.

## Results

### Quantification of biofilm-forming capacity

**Fig. 1:**
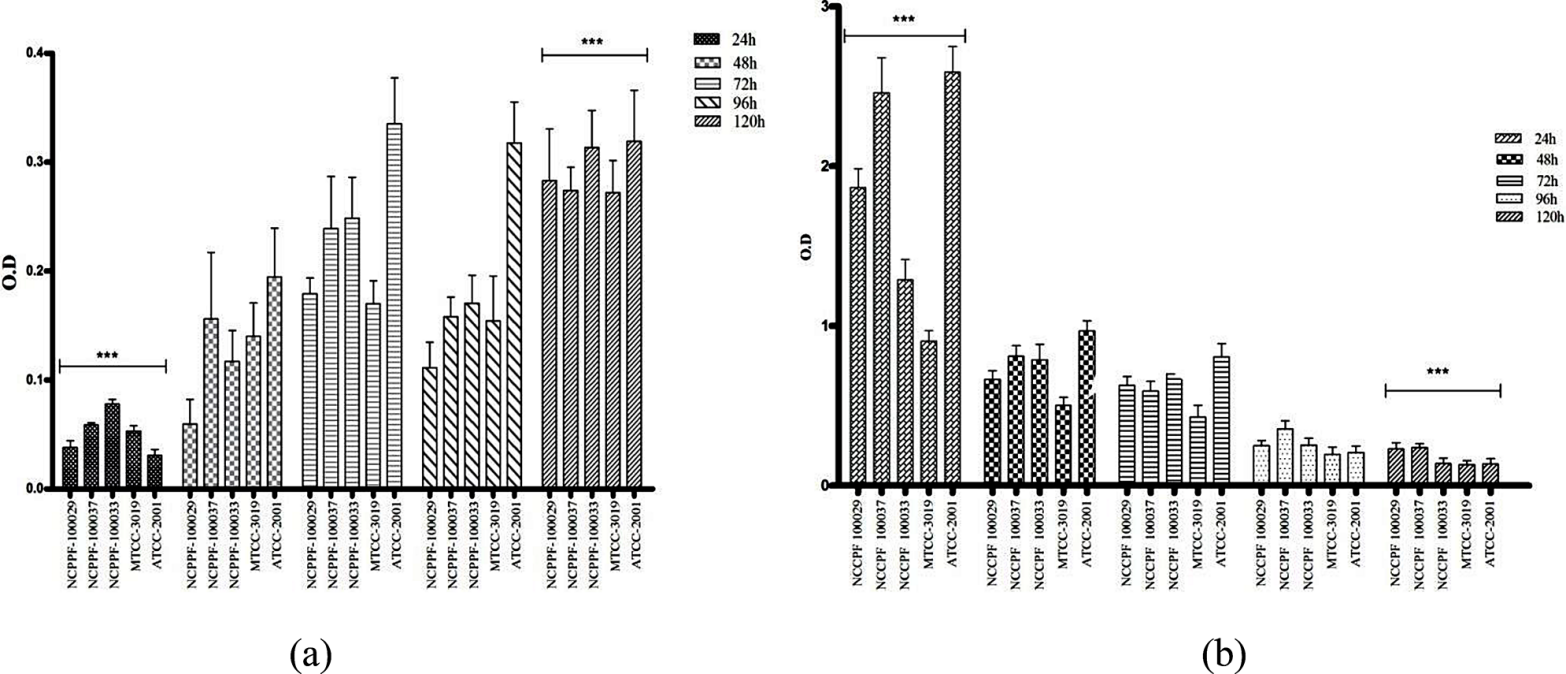
Comparative evaluation of the biofilm forming potential of five different strains of *C. glabrata* assessed by the crystal violet and XTT assay: **(a)** Comparative biomass formation by five strains of *C. glabrata* evaluated using crystal violet assay, all strains showed an increasing trend of biomass up to 120 h. X-axis represents the time of incubation up to 120 h of all strains under investigation, and Y-axis represents the optical density (OD) values taken at 690 nm **(b)** Metabolic activity of cells in the biofilm growth phase of all the tested *C. glabrata* strains evaluated using XTT assay and monitored for 120 h at different time points showed a decreasing trend of OD in all the strains. The ODs presented are the mean +/- standard deviation of three individual ODs evaluated in triplicate with *p* value < 0.05. Bar graph were drawn using GraphPad Prism (v 5.01)

The biofilm forming capacity of all the tested strains of *C. glabrata* (clinical iso- lates NCCPF-100,029, NCCPF-100,033, NCCPF-100,037, standard strains ATCC-2001 & MTCC-3019) revealed that *C. glabrata* (ATCC-2001) has the maximum biofilm formation potential, followed by the clinical isolate NCCPF-100,037, in terms of biomass and metabolic activity (Fig. 1). The total biomass of all strains, measured with crystal violet (CV), showed an increasing trend up to 120 h (Fig. 1a) with *C. glabrata* (ATCC- 2001) strains producing the highest biomass in the biofilm growth phase. The evaluated metabolic activity with 2,3-Bis-(2-Methoxy-4-Nitro-5-Sulfophenyl)-2 H-Tetrazolium-5- Carboxanilide) (XTT) was observed maximum at 24 h for all the tested strains and then gradually decreased up to 120 h. Among all, *C. glabrata* (ATCC-2001) and *C. glabrata* (NCCPF − 100,037) displayed maximum and almost comparable metabolic activity up to 120 h (Fig. 1b).

### Microscopic examination of *C. glabrata* biofilm

**Fig. 2:**
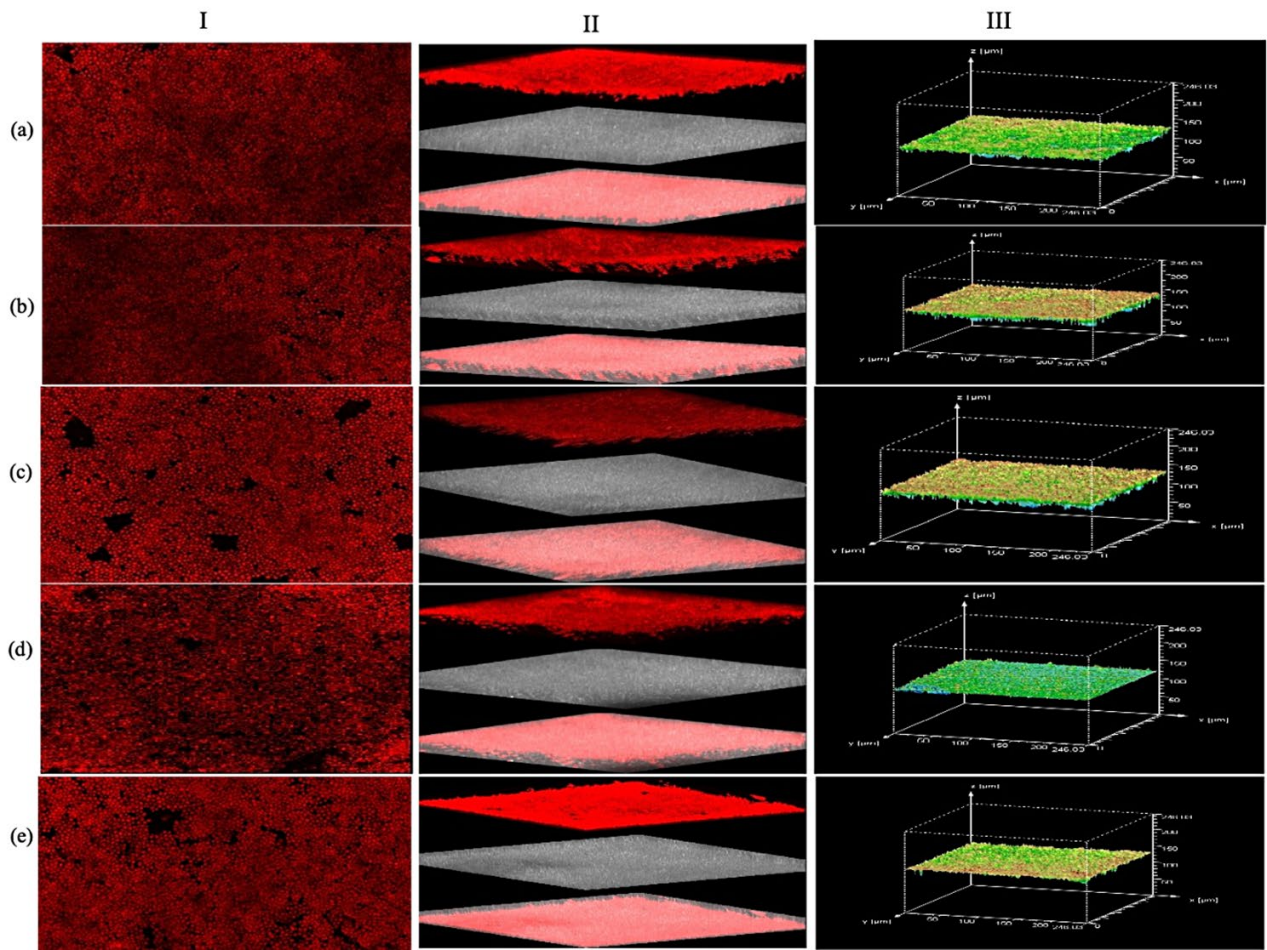
CLSM photomicrographs of biofilm growth phase of five strains of *C. glabrata*: **(a)** ATCC-2001; **(b)** MTCC-3019; **(c)** NCCPF-100,037; **(d)** NCCPF-100,033; **(e)** NCCPF-100,029. Panel I (a, b, c, d and e) represents 1-D CLSM images of *C. glabrata* strains ATCC-2001, MTCC-3019, NCCPF- 100,037, NCCPF-100,033 & NCCPF-100,029, respectively. Panel II (a, b, c, d, and e) represents the images clicked through different contrast and sections to show the overall morphology and architecture of biofilm of all strains in a similar order. Panel III (a, b, c, d, and e) represents one stack out of many stacks captured and a side view reconstructed by Leica application suite (LAS − X) software to visualize the 3-D projection of biofilm thickness of all strains observed

Biofilm morphology and architecture of all five strains was visualized with both confocal laser scanning microscopy (CLSM). It was interesting to observe that comparative analysis of 1-dimensional (1- D) CLSM photomicrographs of all strains exhibited a similar pattern of fluorescent red cylindrical intravacuolar structures (CIVS) with equal fluorescence stained with FUN-1[2chloro4(2,3 dihydro-3-methyl (benzo-1,3-thiazol 2-yl)-methylidene)1 phenyl quinolinium iodide]. Dense CIVS in red color reflects the viability of cells, this indicates that metabolically active cells are present in the biofilm phase of all strains (Fig. 2I, a-e). Comparative investigation of 3-D reconstructed projection of CLSM biofilm images at different contrasts and angles for all strains showed that they also possess similar red fluorescence filaments (Fig. 2 II, a-e). Furthermore, the vertical (x, z) section or side views of the reconstructed 3-D image revealed that all strains exhibited almost same thickness and architecture of biofilm and 3-D projection images with z stacks also showed that they have a biofilm thickness of around 250 µM (Fig. 2 III, a-e).

### RNA-sequencing and analysis of DEGs

Biofilm formation quantification assays and microscopic visualization revealed that *C. glabrata* (NCCPF-100,037) is the best biofilm formers among clinical isolates and its biofilm formation ability is comparable to that of standard strain *C. glabrata* (ATCC-2001). Therefore, the clinical isolate *C. glabrata* (NCCPF-100,037) was used for subsequent experiments such as RNA sequencing, RT-qPCR validation and functional genomics to identify the target genes associated with biofilm formation in *C. glabrata* (NCCPF-100,037). The concentration, quality and integrity of total RNA isolated from all replicate of planktonic (planktonic-I, II&III) and biofilm (24 h) (biofilm-I, II &III) growth conditions of selected clinical isolate *C. glabrata* (NCCPF-100,037) were found to be suitable for the preparation of cDNA libraries and RNA-sequencing (appendix- B). The quality of RNA in terms of the RNA integrity number (RIN) of all biological replicates of both growth conditions (planktonic and biofilm) was found to be 9.8, 9.9 7.7, 9.6, 9.5 & 9.8 in planktonic-I, II, III, biofilm-I, II & III, respectively, (Supplementary Figs. S1, S2). The average GC content of all the sequenced samples was found to be 44.98% and alignment percentage of clean read generated from biofilm-I, biofilm-II, biofilm-III, planktonic-I, planktonic-II, and planktonic-III using TopHat-2 over reference genome of *C. glabrata* was observed 76.42, 76.24, 76.42, 74.38, 68.06 & 86.6% ,respectively, listed in, (Supplementary Table (Supplementary Table S3). The entire high-throughput transcriptomic sequence data of six samples was investigated to identify differentially expressed genes (DEG) in the mature biofilm growth phase of *C. glabrata* (NCCPF-100,037). The expression features detail of differentially expressed genes (DEGs) under both growth conditions such as gene ID, gene locus, normalised expression count in terms of FPKM values, *p*-value, adjusted *p*-value (FDR), transcript ID, transcription start site IDs, class codes, gene annotation, encoded protein, gene ontology annotation of significantly expressed top 50 up-regulated and down-regulated genes is presented in the, (Supplementary Table (Supplementary Table S4), respectively. The raw read files of RNA-Seq and the expression matrix features of the study were submitted to the National Centre for Biotechnology Institute Gene Expression Omnibus database (NCBI-GEO) with project ID: PRJNA640406. Expression analysis obtained a significant 959 (almost 18%) DEGs out of total 5294 genes from *C. glabrata* genome. The whole transcriptomic data employing after the cut-off value of +/- 1.5 to the log2FC (+ 1.5 for up-regulated & -1.5 for down-regulated) with adjusted p-value or (FDR) of < 0.05 resulted into a 363 (6.85%) up-regulated and 596 (11.26%) down-regulated genes respectively under mature biofilm growth phase.

### Visualization of the RNA-Seq data

**Fig. 3:**
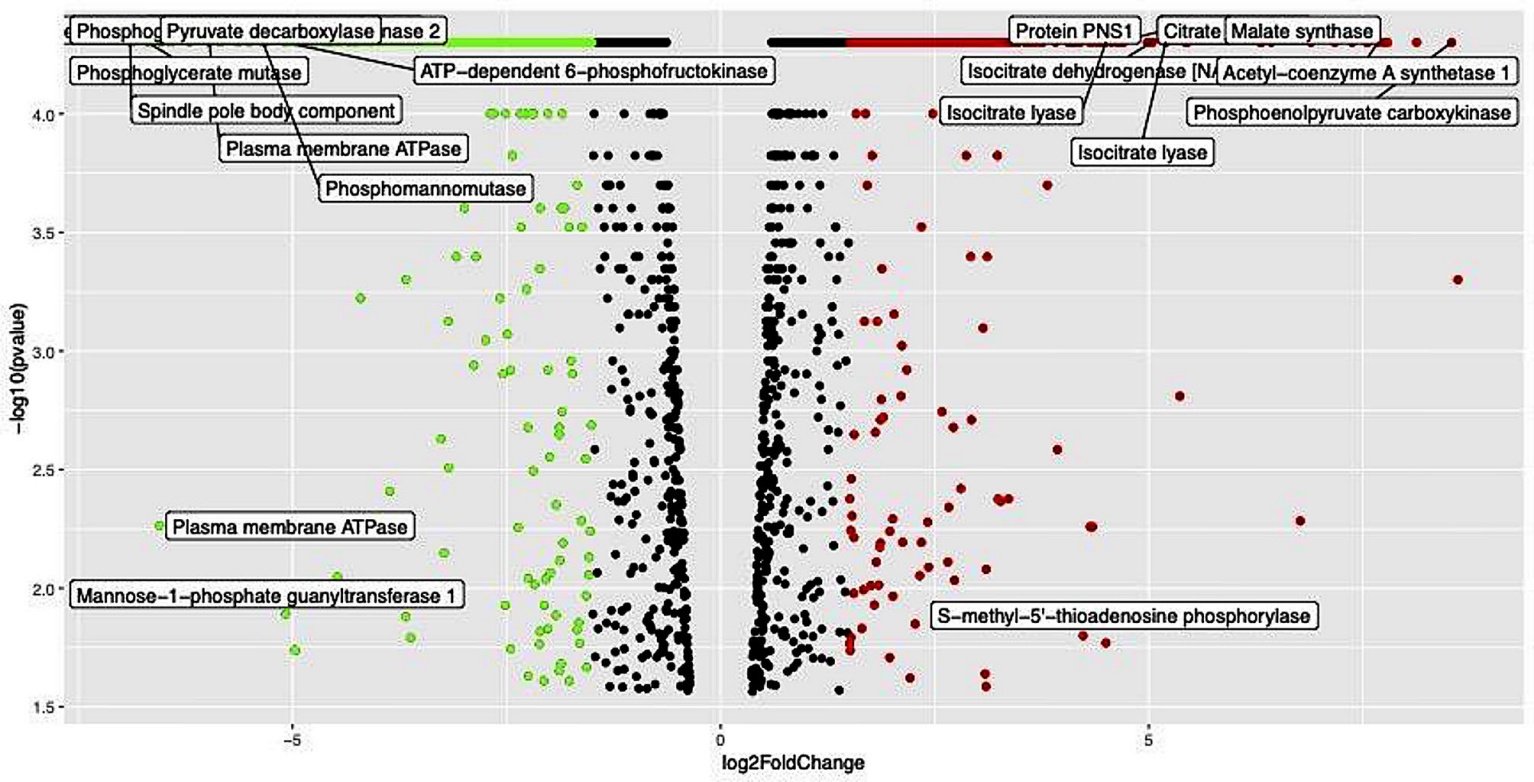
Volcano plot of the DEGs in planktonic and biofilm growth phase of *C. glabrata*. DEGs under individual experimental conditions (planktonic and biofilm) of *C. glabrata*. The X- axis represents the normalised expression of genes in terms of log2 fold change (*log*_*2*_FC), and dots in green and red color demonstrate the significant downregulated and upregulated genes respectively. Y- axis represents statistical significance (*p*-value)

**Fig. 4:**
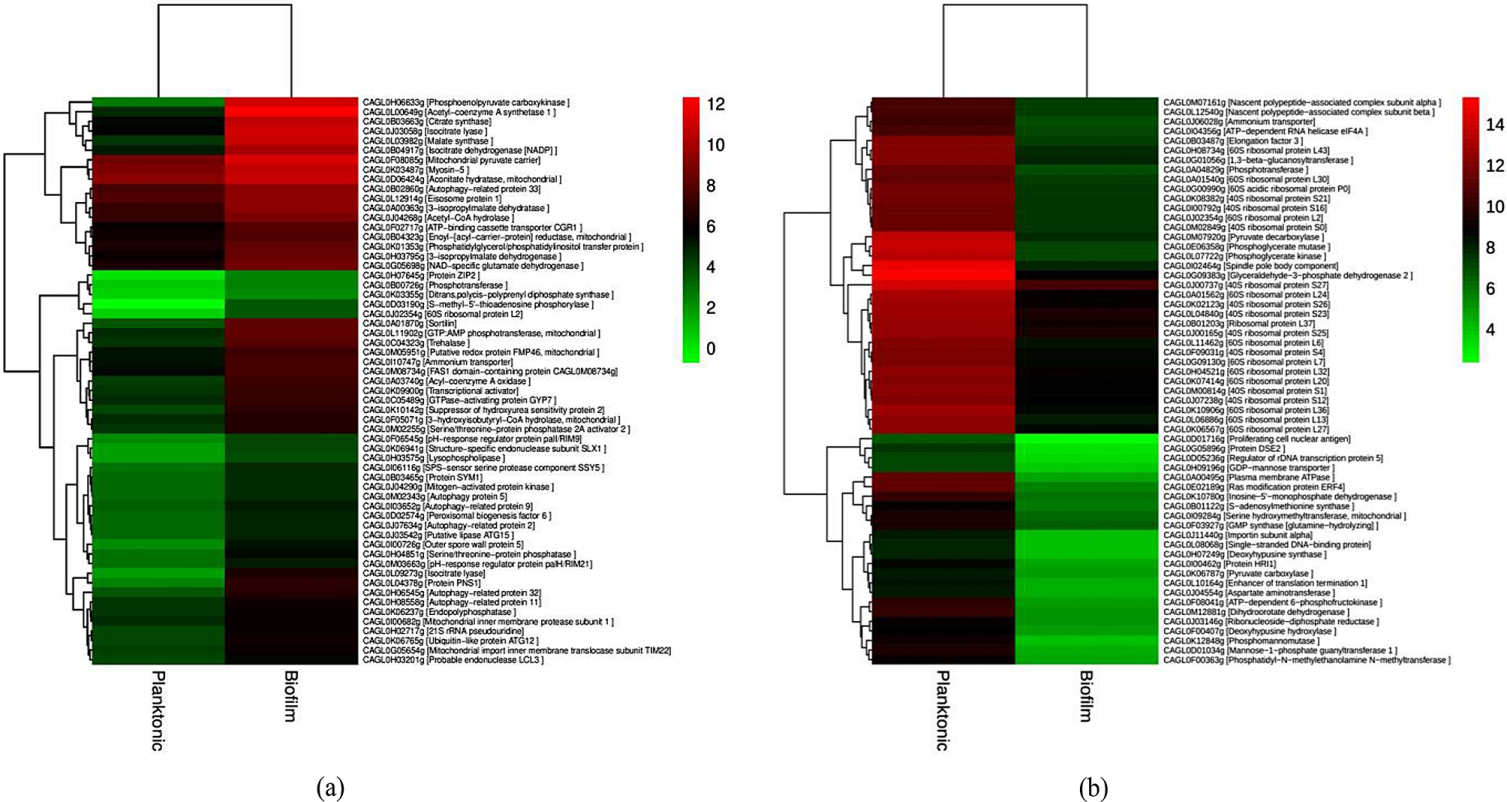
Hierarchical clustering significant DEGs in biofilm growth mode: Shows the top significant up-regulated genes, columns represent the planktonic and biofilm growth condition of *C. glabrata*(Fig. 4a**)** similarly the up-regulated genes are shown in **(**Fig. 4b**)** .Expression of the significant DEGs (listed on the right side of the columns along their encoded proteins) plotted according to their normalized log2FC (FPKM) values in z-scores. Gene expression values of different DEGs are presented in color key code of heatmap with a scale bar of with a heat map of a color scale bar 0–14

Gene expression measured in terms of FPKM values (normalized gene expression ) in both conditions (planktonic vs. biofilm) of *C. glabrata* plotted with a scatter plot revealed that there is a large and significant variation of gene expression under mature biofilm growth phase compared to planktonic condition (Supplementary Fig. S3). The variation in a global transcriptomic landscape under mature biofilm growth phase of *C. glabrata* was depicted in graphical visualization with volcano plots (Fig. 3 & Supplementary Fig. S4). DEGs calculated in terms of log2 fold change (*log*_*2*_FC) FPKM values between two distinct growth conditions (biofilm and planktonic) were plotted against the p- value (-log10 *p*-value). The red and green dots of the volcano plot represent the most significant up-regulated genes and down-regulated genes with high *log*_*2*_FC of FPKM values along with their statistical significance (very low *p*-value). This suggests, that genes such as *CAGL0H010076g*, *CAGL0M07920g* play playing a potential role in the biofilm lifestyle of *C. glabrata*. The list of top significant DEGs with *log*_*2*_FC (1.5 for up-regulated & -1.5 for down-regulated) and normalized z − scores of both growth conditions were visualized using heatmap (Fig. 4). The *log*_*2*_FC values under the two conditions (biofilm and planktonic phase) were presented in hierarchical clusters along with their adjusted *p*-values (FDR). Columns of the heatmap grid representing the growth condition (biofilm and planktonic) and rows represent the differentially expressed genes, whereas the color intensity of the rows displays the expression level across the two conditions (planktonic and biofilm). Gene IDs along with their characterized or putative protein encoded by the respective genes are also shown on the right side of the heat map, and the left side shows the co-expression level dendrogram of the genes.

### Gene set enrichment and pathways analysis of DEGs

**Fig. 5:**
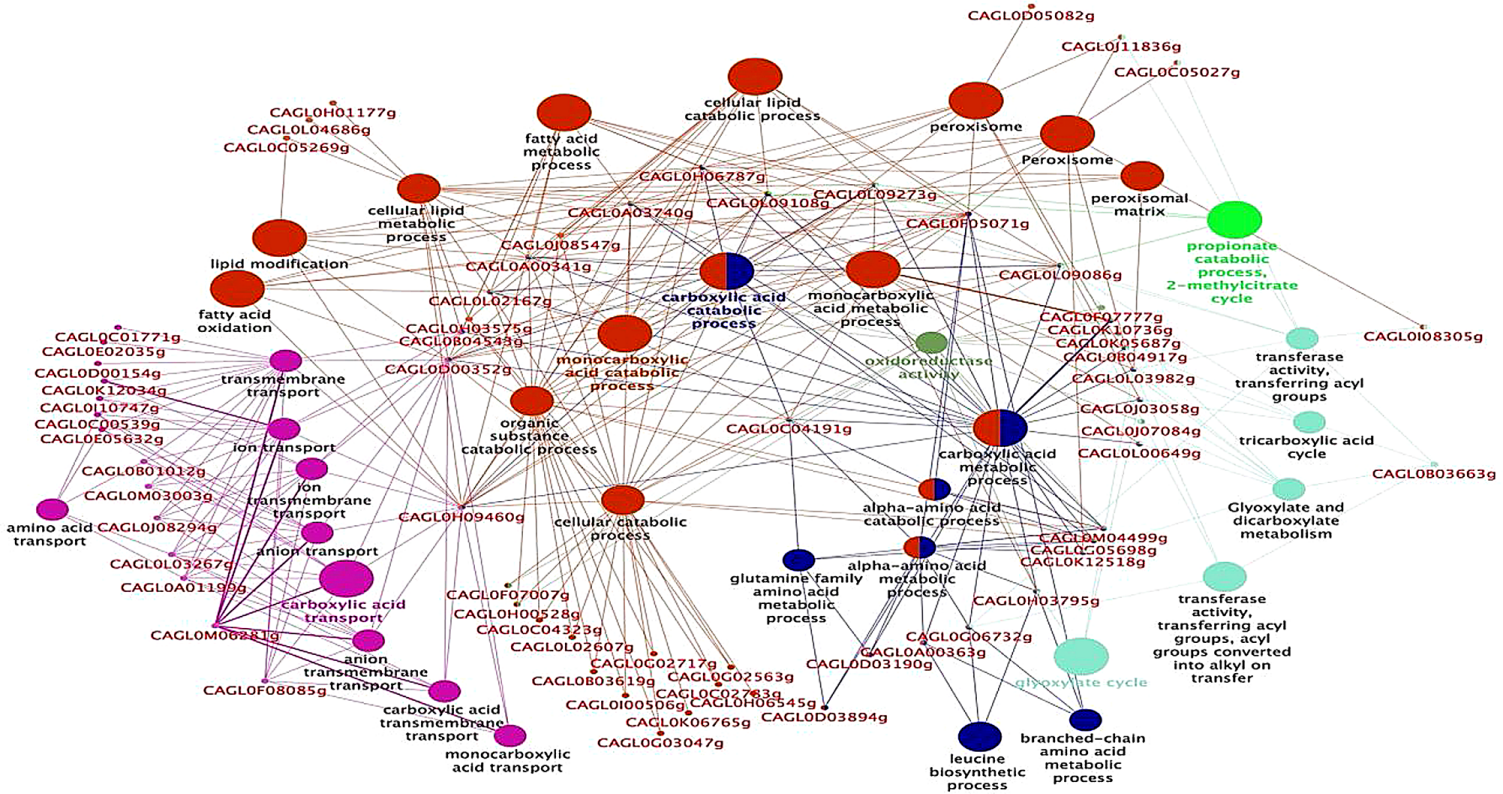
**KEGG pathway network of up-regulated genes in the biofilm growth phase of a clinical isolate of*C. glabrata***: Network analysis of the GO and KEGG pathway of highly significant top upregulated genes involved in the biofilm formation of *C. glabrata*. Pathways depicted by nodes and edges shared by common genes with kappa score 0.4 and *p*-value < 0.05 are displayed with respective colors representing the BP, CC, and MF of the significantly upregulated genes associated with the biofilm growth phase of *C. glabrata*

RNA-Seq data was subjected to elucidate functional enrichment analysis of significantly DEGs, based on their gene ontology and pathways involved in the mature biofilm growth phase of *C. glabrata* (NCCPF-100,037). The most significant list of up-regulated and down-regulated genes were processed separately with ClueGO for analysis of the gene ontology (GO) /Kyoto Encyclopedia of Genes and Genomes (KEGG). The functional annotation enrichment analysis revealed that enriched up- regulated genes are involved in the cellular carbohydrate metabolism, depicted in green color nodes with a molecular function such as glyoxylate and dicarboxylate metabolism, carbon-carbon lyase activity, monocarboxylate, tricarboxylate, citrate metabolism, precursor metabolite generation and energy generation in biofilm cells (Fig. 5). Genes in red color nodes are involved in the cellular catabolic processes of a cell that are responsible for the regulation of autophagy, autophagy, pre-autophagosomal structure, pre-autophagosomal structure membrane, vacuole and vacuolar part. Similarly, orange color nodes depict the genes involved in mitochondrial inner membranes and organelle envelope, whereas pink color nodes demonstrate the fatty acid and lipid metabolic process of peroxisomes. The percentage of genes associated with their biological terms were represented graphically (Supplementary Fig. S5). The names of *C. glabrata* genes associated with biofilm formation are shown in red on the network pathways and the size of the nodes on the network pathways corresponds to the statistical significance of biological terms.

### Verification of DEGs

**Fig. 6:**
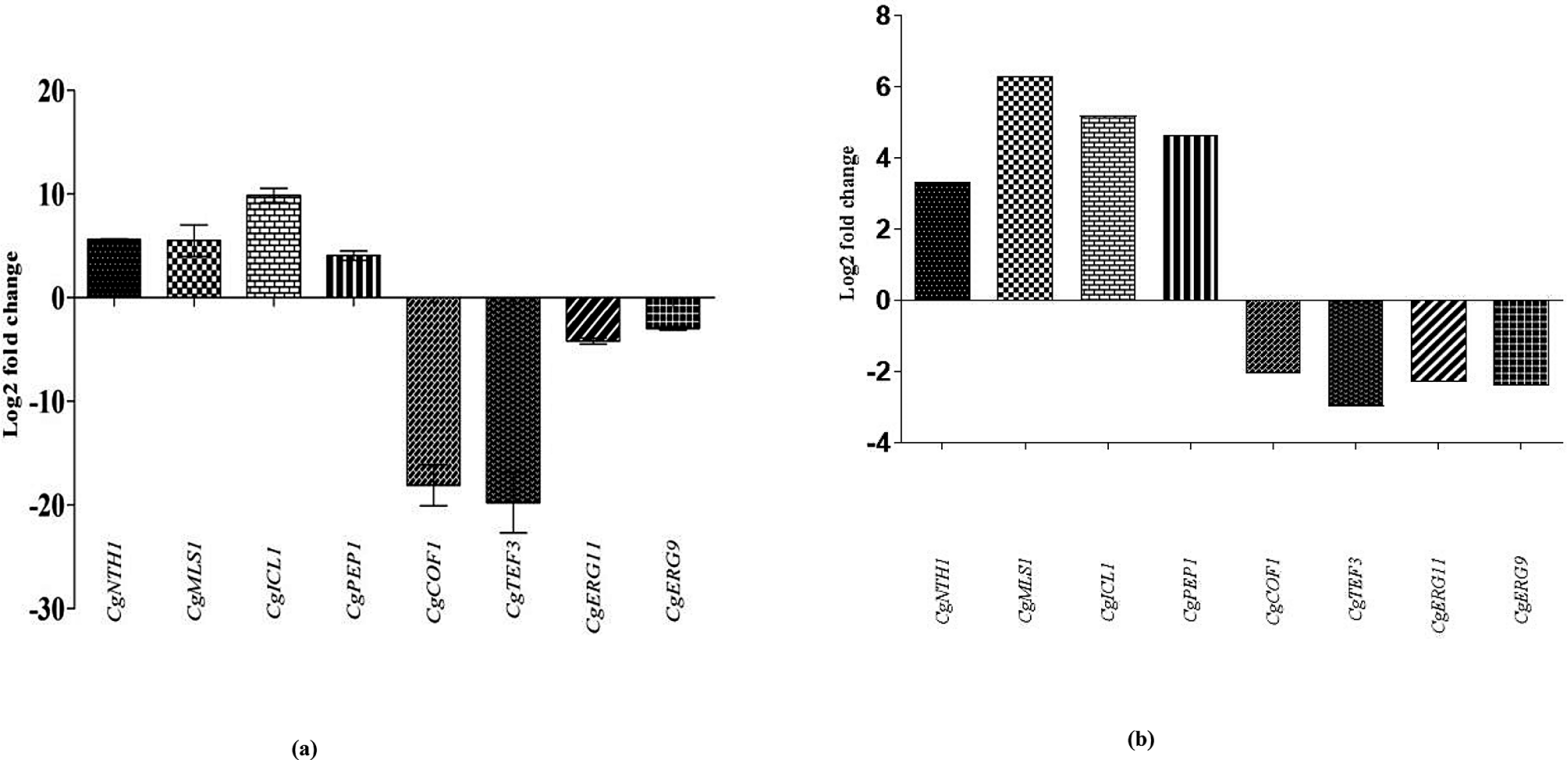
Verification of RNA-Seq expression of DEGs with RT-qPCR:The differential expression of selected genes (Cg*MLS1*, Cg*ICL1*, Cg*PEP1*, Cg*NTH1*, Cg*COF1*, Cg*TEF3*, Cg*ERG9*, and Cg*ERG11*) verified using RT-qPCR. (a) Shows RT-qPCR expression of up-regulated (Cg*MLS1*, Cg*ICL1*, Cg*PEP1*, and Cg*NTH1*) and down-regulated (Cg*COF1*, Cg*TEF3*, Cg*ERG9*, and Cg*ERG11*) genes, quantified in terms of log2FC ∆∆C_T_values.(b)Shows the RNA-Seq expression profile of the same genes, measured terms of log2FC of FPKM values. The bars represented are the mean of three individual fold change value evaluated in triplicate with calculated +/- standard deviation at *p* value < 0.05. Bar graph has been drawn using GraphPad Prism (v 5.01)

The expression verification of eight selected DEGs (Cg*ICL1*, Cg*MLS1*, Cg*PEP1*, and Cg*NTH1*, Cg*ERG9*, Cg*ERG11*, Cg*TEF3*, and Cg*COF1*) was performed with quantitative real-time PCR (RT-qPCR). The gene expression of selected DEGs with RT-qPCR displayed a similar pattern of expression as observed in RNA-Seq. Cg*ICL1*, Cg*MLS1*, Cg*- PEP1*, and Cg*NTH1* genes showed up-regulated expression while the Cg*ERG9*, Cg*ERG11*, Cg*TEF3*, and Cg*COF1* genes were down-regulated (Fig. 6). This validates that the transcriptomic landscape quantified with RNA-Seq and RT-qPCR followed a similar trend of up-regulation and down-regulation in the mature biofilm growth mode of *C. glabrata* (NCCPF-100,037).

### Functional characterization of selected genes

**Fig. 7:**
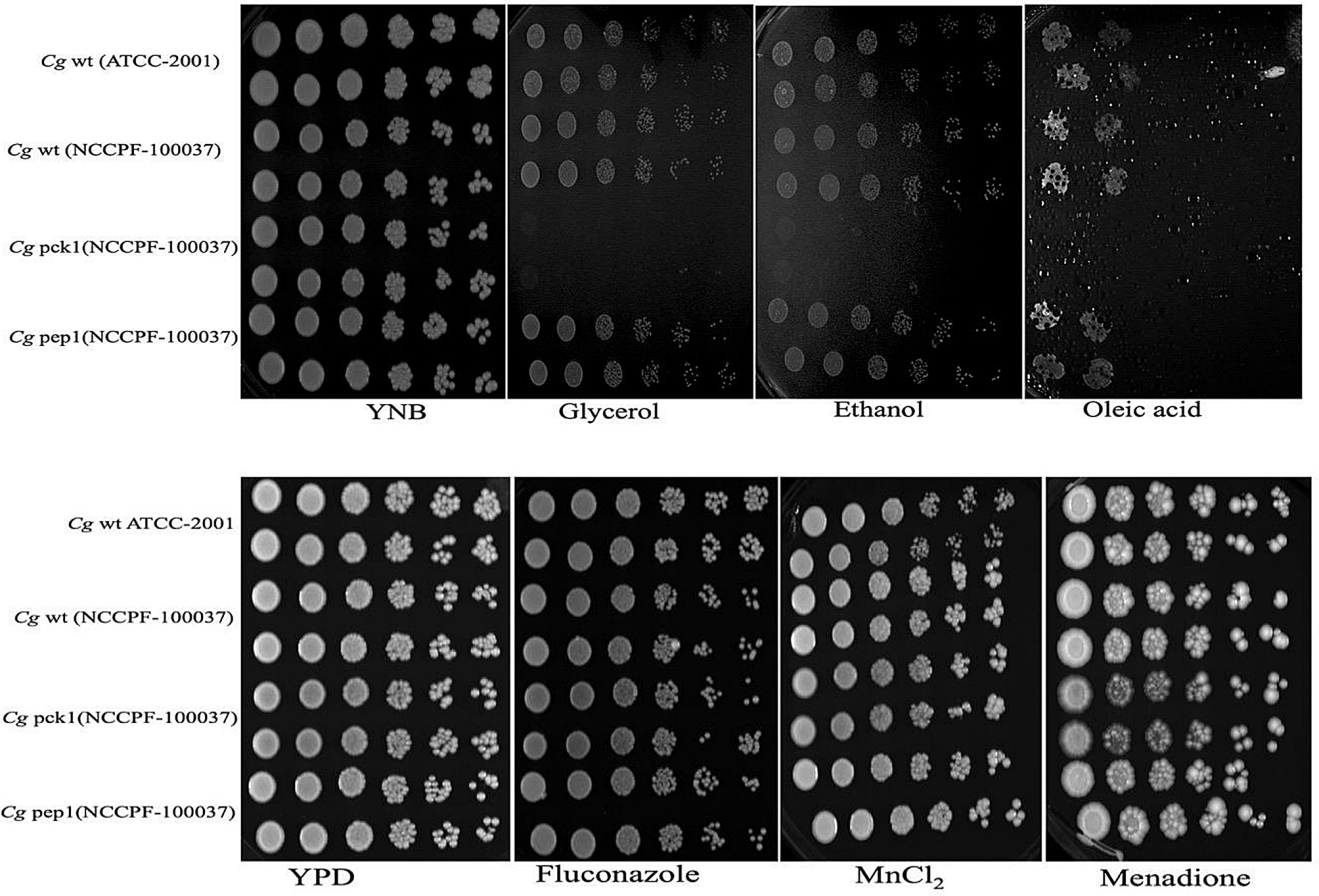
Phenotype profiling of mutant and wild strains of***C. glabrata*on different carbon substrates and under tested stressors**: The overnight grown cultures of standard strain Cg*wt* ATCC-2001, wild type clinical strain Cg*wt* (NCCPF-100,037), and mutant strains Cg*pck1*∆(NCCPF-100,037) Cg*pep1*∆ (NCCPF-100,037) were spotted over YNB with glucose 2%, glycerol 3%, ethanol 2%, and oleic acid 2%. YPD media with fluconazole-16 µg/ mL, 8 mM, manganese chloride (MnCl_2_) – 3 mM, cell-wall stressor for yeast cell menadione − 16 µg/mL). Images of plates were captured after incubation of 24–72 h

**Fig. 8:**
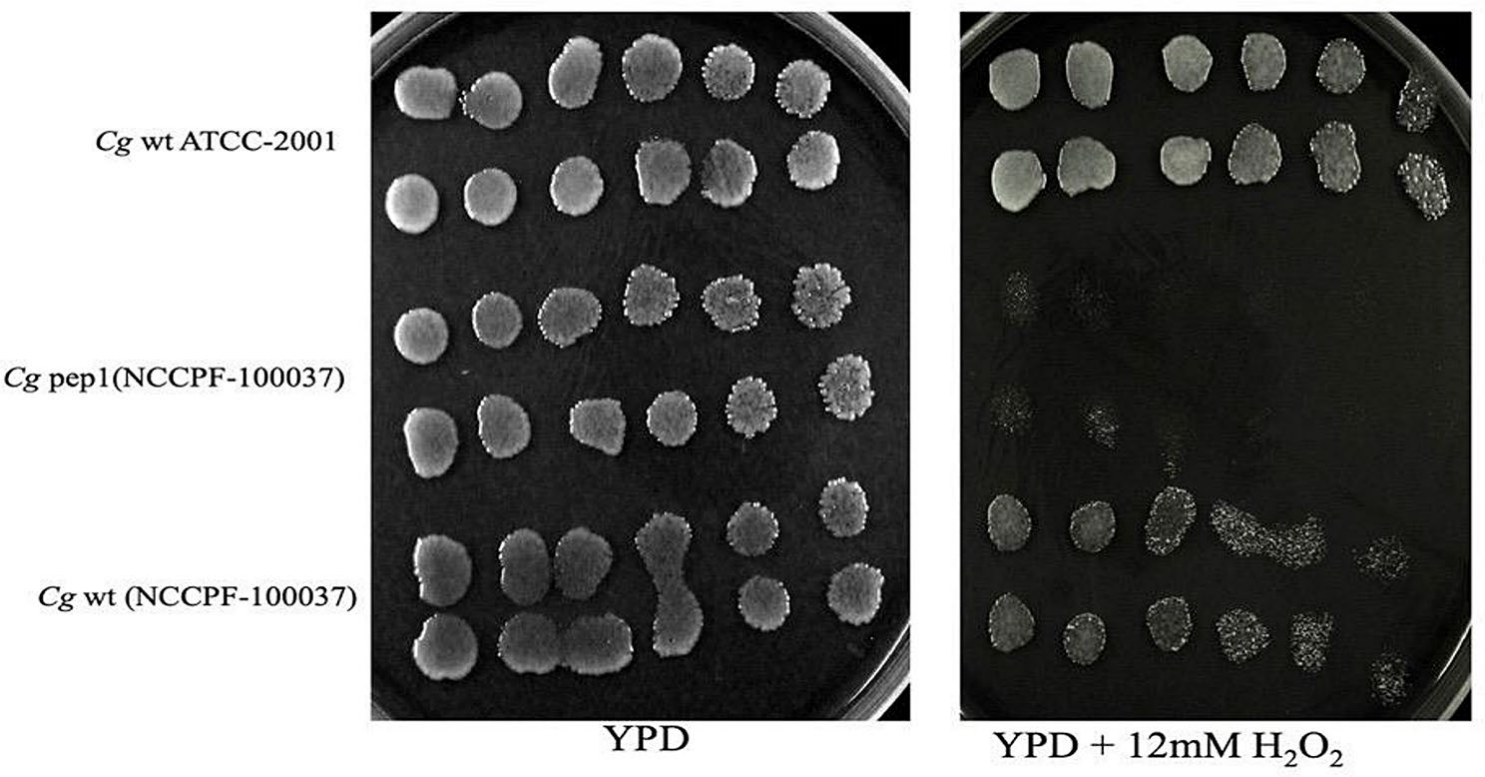
**Phenotype profiling of mutant and wild strains of*C. glabrata*under oxidative stress**: The overnight grown cultures of standard strain Cg*wt* ATCC-2001, wild type clinical strain Cg*wt* (NCCPF-100,037), and mutant strains Cg*pck1*∆ (NCCPF-100,037) Cg*pep1*∆ (NCCPF-100,037) were spotted over YPD + 12 mM hydrogen peroxide. Images of plates were captured after incubation of 24–72 h

i. **Cassette construction** :The deletion cassette for the Cg*PCK1* gene was constructed with 5’ and 3’ untranslated regions (UTR) of Cg*PCK1* gene of *C. glabrata* (NCCPF-100,037) and *Nat1* gene of pRK625 plasmid. The 5’UTR PCR amplification of the Cg*PCK1* gene from wild-type genomic DNA of *C. glabrata* (NCCPF-100,037) resulted into a product of size 641 bp on agarose gel (1.5%), displayed in lanes 1–8 & 10 (Supplementary Fig. S6a). Similarly, an amplified PCR product of 654 bp was observed from 3’UTR of the Cg*PCK1* gene of *C. glabrata* (NCCPF100037). These two amplified PCR products from 5’ and 3’ UTR of Cg*PCK1* were fused one by one with each half of the amplified *Nat1* gene of pRK625 plasmid and generated two fusion PCR products FP1 and FP2 of size 1403 bp and 1519 bp respectively (FP1-1403 bp = 5’ *Nat1* + 5’UTR region of a Cg*PCK1* & FP2-1519 bp = 3’ *Nat1* + 3’UTR region of Cg*PCK1*) in lanes 1 and 2, where M is the marker of 1 kb (Supplementary Fig. S6b). The fusion PCR products FP1 and FP2 share 310 bp complementary regions to each other as it is amplified from the *Nat1* gene. Therefore a mixture of FP1 and FP2 served as a deletion cassette and was used to knockout the Cg*PCK1* gene from wild- type *C. glabrata* (NCCPF-100,037) with a homologous recombination process. Similarly, the disruption cassette for knocking out the Cg*PEP1* gene from a wild-type *C. glabrata* (NCCPF-100,037) was synthesized with FP1 and FP2 of size 1377 bp and 1490 bp. The PCR amplification of 5’&3’UTR of the Cg*PEP1* gene from wild-type genomic DNA of *C. glabrata* (NCCPF100037) produced gene products of 615 bp (lanes 1–9) and 625 bp (lanes 10–16), respectively, observed on agarose gel (1.5%) (Supplementary Fig. S7a). The fusion PCR of these products one by one with amplified *Nat1* gene generated FP1 and FP2 of size 1377 bp (lane-1) and 1490 bp (lane-2) observed on agarose gel (1.5%) (Supplementary Fig. S7b).
ii. **Verification of genes (Cg*PCK1*and Cg*PEP1*) disruption in*C. glabrata*(NCCPF-100,037)**: PCR amplified product with internal primers for target gene Cg*PCK1* demonstrated the absence of this gene in genomic DNA isolated from the transformant mutant strains *C. glabrata* (NCCPF-100,037 or Cg*pck1*∆). There was no amplification observed (of Cg*PCK1* gene) in lanes 5–13, and lane-1 represent the marker of 100 bp (Supplementary Fig. S8). Whereas, PCR product of size 334 bp were amplified with internal primer from genomic DNA of wild type *C. glabrata* (NCCPF-100,037) strain in lanes 2&4. Further, PCR amplification of full *Nat1* cassette from genomic DNA of *C. glabrata* (NCCPF-100,037) mutant (transformants) strain confirmed its insertion in place of target knocked-out Cg*PCK1* gene. PCR amplification with expected product size of full *Nat1* cassette (547 bp) was observed in the lanes (2, 4, 6, 8, 9, 10 and 11) from genomic DNA of *C. glabrata* (NCCPF-100,037) mutant strains (transformants), conversely there was no amplification observed in no template control (NTC) (lane-12) and wild-type genomic DNA template (lane-13) (Supplementary Fig. S8).This is verified that the cassette has been inserted exactly in the place of target gene and resulted in a knock-out strain of *C. glabrata* (NCCPF-100,037) for Cg*pck1*∆ strain. There was no amplification observed from the genomic DNA of wild type *C. glabrata* (NCCPF-100,037) in lane-13, lane-1 show the molecular marker M of size 100 bp (b) The lane-2, shows the PCR amplified product of a size 302 bp using internal primer for gene Cg*PEP1* with genomic DNA from wild type *C. glabrata* (NCCPF-100,037) and taken as a positive control. Lanes 4–17 showed no PCR amplification using internal primer for gene Cg*PEP1* with genomic DNA isolated from the Cg*pep1*∆ mutant (transformants), conversely lane-3 showed PCR amplified product of a size 302 bp with Cg*pep1*∆ mutant DNA. Similarly, PCR with internal primers for target gene Cg*PEP1* from the genomic DNA isolated from the transformant mutant strains *C. glabrata* (NCCPF-100,037 or Cg*pck1*∆ showed no amplification in lanes 4–17 (Supplementary Fig. S9a). Whereas, genomic DNA from wild type strains of *C. glabrata* (NCCPF-100,037) showed the amplification of PCR band of size 302 bp with internal primer for Cg*PEP1* gene (Supplementary Fig. S9b). Additionally, genomic DNA from mutant strains (transformant) of *C. glabrata* (NCCPF-100,037) showed amplification of full *Nat1* cassette in lanes 2–11 (Supplementary Fig. 9b).whereas, DNA from wild of the same strain displayed absence of any PCR band in lane 13 confirms the knocking-out of Cg*PEP1*(Supplementary Fig. S9).
iii. **Phenotype screening of knockout strains**: The knocking out of genes was followed by the functional validation of Cg*PCK1* and Cg*PEP1* in response to the nutritional limitations and stresses. Growth phenotype of *C. glabrata* strains lacking Cg*PCK1* and Cg*PEP1* genes was observed on alternative carbon sources such as glycerol, ethanol and linoleic acid). Growth and viability of mutants strains were also checked in the presence of antifungal agent (fluconazole), oxidative stress inducers (menadione, H_2_O_2_) and in metal ion stress (MnCl_2_) conditions. Analysis of growth phenotype screening revealed that Cg*pck1*∆ mutant failed to grow on yeast nutrients broth (YNB) plates supplemented with alternative carbon sources such as glycerol, ethanol and linoleic acid. Furthermore, its growth and viability remained unaltered in the media in presence of fluconazole, metal stress inducer (MnCl_2_) and oxidative stressors such as menadione and hydrogen peroxide H_2_O_2_ (Fig. 7). This suggested that Cg*pck1*∆ mutant unable to utilize the carbon substrates other than glucose such as glycerol, ethanol and linoleic acid. On the other hand, Cg*pck1*∆ mutant could withstand the fluconazole, metal stress and oxidative stressors. Furthermore, Cg*pep1*∆ showed growth on alternative carbon substrates (glycerol, ethanol and linoleic acid), fluconazole and stress inducers such as menadione and (MnCl_2_) but it failed to grow in presence of H_2_O_2_(Fig. 8). This indicates that Cg*PEP1* is associated with oxidative stress in the clinical isolate *C. glabrata* (NCCPF-100,037).

## Discussion

Metabolic plasticity prevailing in biofilm growth phase of *C. glabrata* needs to be unraveled in order to understand its pathobiology and designing novel antifungal regimen to tackle infections caused by this pathogen. The observations of biofilm forming assays (qualitative, quantitative) and microscopic analysis (morphology and architecture) of biofilm cells indicated that among all the three clinical strains of *C. glabrata* (NCPPF-100,037, NCPPF-100,033, NCPPF-100,029), *C. glabrata* (NCCPF-100,037) is a strong biofilm former and its biofilm capacity is almost similar to that of the standard strain of *C. glabrata* CBS-138 (ATCC-2001), which has been previously reported as a potent biofilm former [20, 40]. Therefore, the present study was designed to identify genes involved in sustaining mature biofilm growth phase of clinical isolate of *C. glabrata* NCCPF-100,037. The clinical strain was preferred over the standard strain under current investigation, because of the following reasons (i) *C. glabrata*-NCCPF-100,037 isolated from a patient with blood-stream infection (BSI)/candidemia and happens to be more virulent after overcoming the host immune defence mechanisms during the host-pathogen interaction, (ii) Standard laboratory strains might have lost the important genes responsible for biofilm formation while repeated culturing in the laboratory [41–43]. Furthermore, a recent study reported significant differences in the surface hydrophobicity, oxidative stress induction and susceptibility to azole based antifungal drug, between clinical isolates and standard strains of *C. glabrata* [44]. This prompted us to use clinical strains of *C. glabrata* for transcriptomics investigation.

The RNA-Seq based transcriptomics approach has been pivotal in cataloguing the landscape of gene structure, profiling transcriptome expression and functional genome characterization under varied conditions of the pathogen [45, 46]. Here, we attempted to demonstrate how differential expression of genes remodel the cells to counter the nutrient and oxidative stress in biofilm growth conditions in *C. glabrata*. In the present study, most of the significant DEGs obtained are uncharacterized and their functions are not verified to date, comprising 72.51% (694) of the total observed DEGs (959). A possible reason for this could be that only 530 (10%) of the total 5271 ORFs of *C. glabrata* have been confirmed and characterized so far, whereas *C. albicans* has 3598 (29%) out of 12,405 ORFs which is nearly three times the former [47, 48]. This suggests that genomics and transcriptomics of *C. glabrata* are understudied and more research into genome annotation and characterization of this opportunistic pathogen is required. The extensive data visualization plots identified and screened significant DEGs out of a large data set that may be involved in the biofilm growth modes [32, 49].

The observations of functional annotation, gene ontology, and pathways analysis pointed out that significant upregulated DEGs are involved in carboxylic acid catabolism, carboxylic acid transport, glyoxylate cycle, monocarboxylic acid catabolism, oxidoreductase activity, propionate catabolic process, and 2-methyl citrate cycle. These observations are in accordance with previous reports where transcriptomics and proteomics findings of *C. glabrata* have suggested that upregulated genes are involved in alternative carbon substrate utilization of stress related proteins and down-regulation of glycolytic pathways under biofilm growth conditions [50–52]. These results indicate that *C. glabrata* experiences various stress conditions and nutritional shortages throughout the mature growth phase. Therefore, expression of stress resistance genes are elevated and those involved in glycolytic pathways are downregulated to save the energy supply. In contrast, transcriptomics and functional gene analysis of high biofilm former *C. albicans* reported the upregulated pathways are associated with amino acid (including arginine, aspartate, glutamate and proline), fatty acid and pyruvic acid metabolism [53–55]. More specifically, verification of the differentially expressed genes and functional characterization confirmed their possible involvement in shaping up the homeostasis of cells in the mature biofilm. Interestingly, in our study, upregulation of the genes (such as Cg*ICL1*, Cg*MLS1*, Cg*PCK1* andCg*NTH1*) within biofilm cells of clinical isolate of *C. glabrata*is associated with carbon metabolism. This suggested that *C. glabrata* encounters a dearth of preferred carbon substrate (glucose) concentration in mature biofilm and to circumvent this, opportunistic pathogen may reprogram its carbon metabolism by various means.

**Fig. 9:**
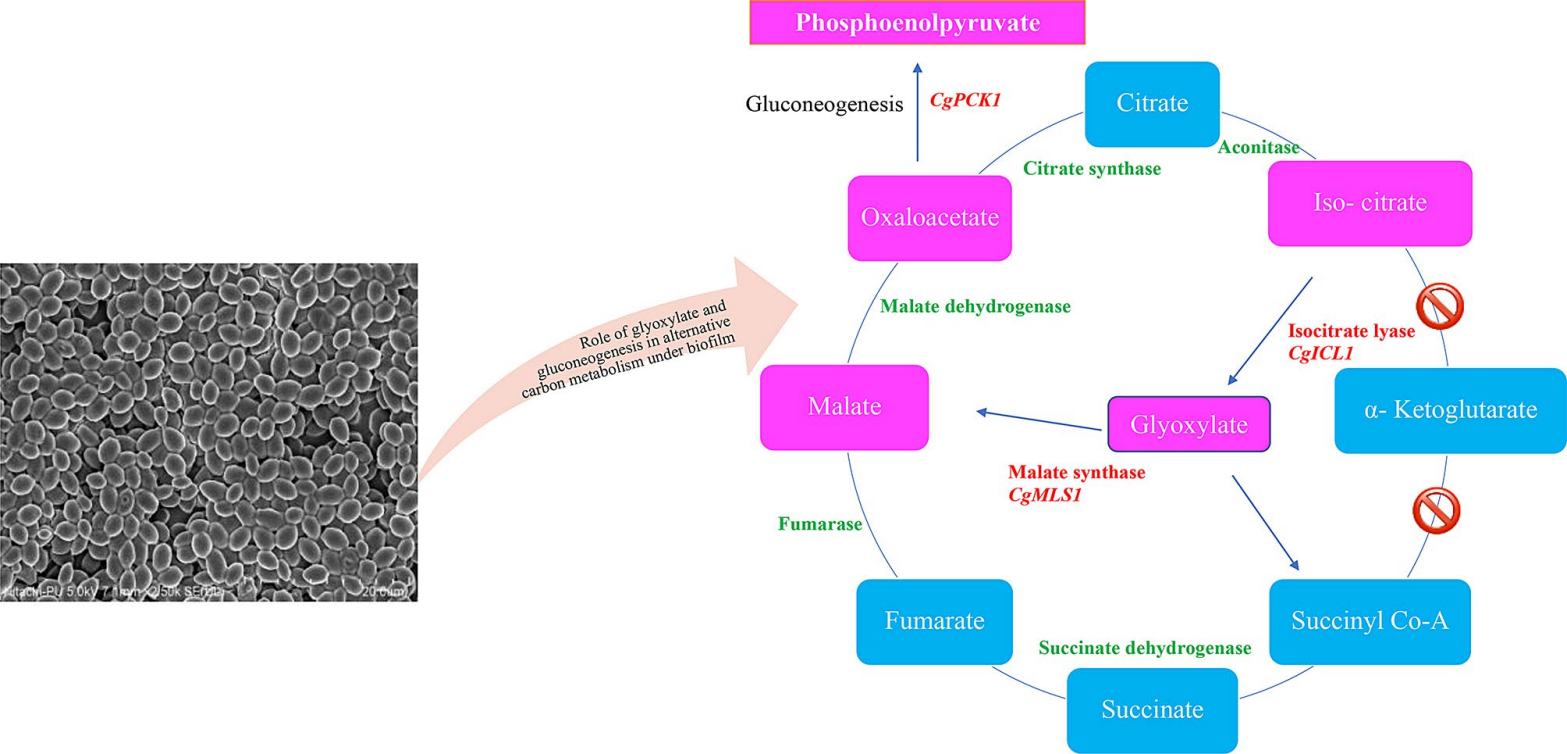
Glyoxylate and gluconeogenesis pathways of carbon substrate metabolism in biofilm growth mode of *C. glabrata*(NCCPF-100,037)

Firstly, it may adopt an alternative glyoxylate pathways instead of tricarboxylic acid cycle (TCA), where glyoxylate is biosynthesized from isocitrate, catalysed by a key enzyme isocitrate lyase (ICL1) and subsequently glyoxylate is converted into malate using malate synthase enzyme (MLS1) (Fig. 9) [50, 52]. These observations suggest that *C. glabrata* bypass the two-carboxylation steps of the TCA cycle (Isocitrate to alpha-ketoglutarate and subsequently to Succinyl CoA) in mature biofilm conditions that enhance its virulence [52, 55, 56]. It is in accordance with earlier studies, where it has been demonstrated that the metabolic plasticity of *C. glabrata* modulates the virulence and survival ability in nutrient-starved condition to counter the stress in a biofilm growth phase and channelizes to promote pathogenicity from commensal to opportunistic mode [57–59]. Secondly, it may follow gluconeogenesis pathway by mediating the upregulated expression of Cg*PCK1*, which has been reported previously in the reprogramming and regulation of carbon assimilation in pathogenic *C. albicans* [60]. Thirdly, *C. glabrata*(NCCPF-100,037) cells may also hydrolyse the trehalose into two glucose units to overcome the glucose scarcity in mature biofilm with enhanced trehalase (NTH1) enzyme expression, which has been reported previously in *S. cerevisiae* [61, 62]. These pathways are interconnected and enhance fitness attributes and metabolic rewiring in the biofilm lifestyle of *C. glabrata*.

After comprehensive analysis of DEGs next we chose two genes (Cg*PCK1* and Cg*PEP1)*, whose expression was significantly elevated under mature biofilm of *C. glabrata* (NCCPF − 100,037). Previous reports have shown that under biofilm growth mode, *C. glabrata* encounters nutrient and oxidative stress conditions [52]. Thus, to confirm their role in resistance towards stresses prevailing in the mature growth mode of *C. glabrata* (NCCPF − 100,037), we knocked-out the respective genes using a cassette deletion approach. Firstly, we investigated whether Cg*PCK1* expression is associated with nutrient stress of the preferred carbon source and how it overcomes this. Phenotype profiling results of our study with Cg*pck1*∆ and wild strain of *C. glabrata* (NCCPF − 100,037) on alternative carbon sources (glycerol, ethanol, and oleic acid) confirmed that the Cg*PCK1* gene is essential to assimilate and metabolize the glycerol, ethanol, and oleic acid. These results suggest that in the absence of glucose, *C. glabrata* assimilate glycerol, oleic acid and generate acetyl coenzyme A (acetyl-CoA) which is a central and connecting metabolite between catabolic to anabolic pathways (glyoxylate and gluconeogenesis) to produce glucose and fulfil energy requirements. The key gene (Cg*PCK1*) which encode the enzyme to Phosphoenolpyruvate (PEP) carboxykinase to produce acetyl coenzyme A (acetyl-CoA) through PEP from oxaloacetate of glyoxylate cycle plays an important role in this carbon assimilation pathways. These findings are in concordance with previous reports where they have reported alternative carbon metabolism in the virulence of *C. albicans* and fitness attribute of *S. cerevisiae* [50, 63, 64]. Thus, our transcriptomic and functional genomic observations showed its overexpression and functional validation in the biofilm of *C. glabrata*. These findings, were also in line with previous observations where overexpression of Cg*PCK1* were documented in the reprogramming and regulation of carbon assimilation in pathogenic *C. albicans* [60]. NMR spectroscopy based metabolic profiling in *C. glabrata* underscores the significant reorganization of carbon metabolic pathways according to the suitable carbon substrate, indicating that this pathogen successfully adapts to varied physiological conditions [65].

In second gene deletion experiment, we knocked-out Cg*PEP1* to examine whether Cg*PEP1* expression is involved in oxidative stress resistance. Here, we assessed the growth phenotype of wild and Cg*pep1*∆ strains of *C. glabrata* (NCCPF − 100,037) with H_2_O_2_and observed phenotype profiling. Growth impairment of Cg*pep1*∆ strains of *C. glabrata* in the presence H_2_O_2_suggested that Cg*PEP1* is involved in countering the ROS stress of H_2_O_2_. Gene deletion of Cg*PEP1* along with phenotype profiling of mutant strain, indicated its role in the degradation and sorting of oxidatively damaged proteins and counter the oxidative stress damage due to hydrogen peroxide in mature the biofilm of *C. glabrata* (NCCPF-100,037) and prevent cell death. This is in accordance with previous study where PEP4 was reported to be associated with oxidative stress in *S. cerevisiae* [66–68]. The current study highlighted that (i) Up-regulation of Cg*ICL*, Cg*MLS* andCg*PCK1* genes are involved in the glyoxylate cycle and gluconeogenesis pathway are up-regulated in the biofilm growth phase (ii) Deletion of (Cg*PCK1 and* Cg*PEP1*) genes, confirmed their role in nutrient and oxidative stress under mature biofilm growth phase of *C. glabrata* (NCCPF100037). Thus, the information obtained from the present study can be concluded that genes Cg*ICL*, Cg*MLS* and Cg*PCK1* are involved in the glyoxylate cycle and could be used as novel antifungal targets to treat candidiasis caused by *C. glabrata*.

## Conclusion

The current study deciphered the important genes, gene sets and their interactome that could play a pivotal in the biofilm formation. Additionally, the gene deletion approach confirmed the role of Cg*PCK1* in alternative carbon source assimilation and Cg*PEP1* in protein sorting to counter the nutrient and oxidative stress prevailing in the mature biofilm of *C. glabrata* (NCPPF-100,037). Thus, the study demonstrated that up-regulation of Cg*ICL*, Cg*MLS* and Cg*PCK1* are associated with the glyoxylate cycle and gluconeogenesis pathways. The observations deduced from the present study along with future work on characterization of the proteins involved in this intricate process may prove to be beneficial for designing novel antifungal strategies.

### Electronic supplementary material

Below is the link to the electronic supplementary material.

Supplementary Material 1

Supplementary Material 2

## Data Availability

All the data has been provided in the manuscript except RNA-Sequencing raw reads data and the same is available in bio project with accession number PRJNA680327; GEO: GSE162024. (https://www.ncbi.nlm.nih.gov/geo/query/acc.cgi? acc=GSE162024).
