# Supplementary material for: Decoding the role of oxidative stress resistance and alternative carbon substrate assimilation in the mature biofilm growth mode of Candida glabrata
Source: BMC Microbiol. 2024 Apr 20;24:128. doi: 10.1186/s12866-024-03274-9 (PMC11031924; doi:10.1186/s12866-024-03274-9)
Supplement: Supplementary file 1 — Supplementary Material 1 [file 12866_2024_3274_MOESM1_ESM.docx]

**Table S1:** List of primers used for DEGs verification with RT-qPCR

| **Primer Name** | **Primer Sequence** | **Primer Size(bp)** |
| --- | --- | --- |
| *CgICL1* | Forward: AATGTGCGCGGACATAATGC | 20 |
|  | Reverse : AAGGGCTCGATATCTGGGTTC | 21 |
| *CgMLS1* | Forward : AAGCCACAATTCGAGCCATC | 20 |
|  | Reverse : GCCTGTTCTCAAGCAATTGC | 20 |
| *CgPEP1* | Forward : TTGTTGCGGAAGACAATGGC | 20 |
|  | Reverse : AATTGCAACCCGGAGCTATC | 20 |
| *CgNTH1* | Forward : AGCAAAAGCATGGCCTTCAG | 20 |
|  | Reverse : AGCCACGTTGTAGAACACAC | 20 |
| *CgERG11* | Forward :TGACCGACCAAGAAATTGCC | 20 |
|  | Reverse : AGACGCGCATTTGTTCTTGG | 20 |
| *CgERG9* | Forward: TGCAGGTTTTGGTTCTGACG | 20 |
|  | Reverse: AACGACCGTCATCCAAATCC | 20 |
| *CgCOF1* | Forward: GCTTTGAAGAGAGCCTTGAACG | 22 |
|  | Reverse: TTAGTGAGAACCAGCACCTCTG | 22 |
| *CgTEF3* | Forward : AACTTCTTGGAAGCCGTTGC | 20 |
|  | Reverse : AAGCAACAGCTTGGACATCC | 20 |
| *Cg18S* | Forward : AGAAACGGCTACCACATCCAA | 21 |
|  | Reverse : CGAATGGGCCCTGTATCGT | 19 |

**Table S2:** List of primers used for mutant generation and gene deletion confirmation

| **Primer Name** | **Primer sequence (5’-3’)** | **Primer length(bp)** |
| --- | --- | --- |
| PCK1 (Internal) | Forward : CCAATGCAAGAACCCAAGAT | 20 |
|  | Reverse : GCGTAACAACCACCTTCGAT | 20 |
| PCK1 5’UTR | Forward : CCGTAGCACCCAAAGAACAA | 20 |
|  | Reverse : gcgtcgacctgcagcgtacgTGCAGTTTCCATCCGCTATT | 40 |
| PCK1 3’UTR | Forward : cgacggtgtcggtctcgtagTGCAAGAACAGAAAAGGCAAT | 41 |
|  | Reverse : ATAATGCCAGCGATGACTGA | 20 |
| Integration check | Forward : AAGTGGAAAAGCCATCGAGA | 20 |
|  | Reverse : TCTTGGCTGTGGTAGGCTCT | 20 |
| PEP1 (Internal) | Forward : GTGATCATGCAGACCGCTAA | 20 |
|  | Reverse : CTTGTACCTGGGATGCCACT | 20 |
| PEP1 | Forward : GCCGATAGTTTACTTGCGGTA | 21 |
| 5’ UTR | Reverse : gcgtcgacctgcagcgtacgGGACTGAAATTTCCCTGACC | 40 |
| PEP1 | Forward : cgacggtgtcggtctcgtagCCCCGCGGATATAGTAATCA | 40 |
| 3‘ UTR | Reverse : TGAAAAGCCCTTGTTGGTCT | 20 |
| Integration check | Forward : CCGTGTCTGTTGAGGGTTTT | 20 |
|  | Reverse : CTGGTGAGTGTGCTCTTGGA | 20 |
| **Nat1 Cassette Primers** | | |
| OGRK-342 Forward | CGTACGCTGCAGGTCGACGCCTTCCGCTGCTAGGCGCGCCGTG | 43 |
| OGRK-343 Reverse | CTACGAGACCGACACCGTCGGGCCGCTGACGAAGT | 35 |
| OGRK-342 Forward | GTCTACTACTTTGGATGATAC | 21 |
| OGRK-343 Reverse | TCTGTTCCAACCAGAATAAG | 20 |
| OGRK-342 Forward | TGCGCACGTCAAGACTGTCAAGG | 23 |
| OGRK-343 Reverse | TGTGAATGCTGGTCGCTATACTGC | 24 |

**Table S3:** Reads mapping summary of all sequenced replicate of both conditions (planktonic- I, II, III and biofilm I, II, III).

| **Sample Name** | **Number of Reads (paired- end)** | **% GC** | **% Q >30** | **QC passed reads** | **QC passed reads %** | **Aligned Read counts** | **Aligned Read counts %** |
| --- | --- | --- | --- | --- | --- | --- | --- |
| Biofilm-I | 24675605 | 45.7 | 90.4 | 23651567 | 95.85% | 18074527 | 76.42 |
| Biofilm-II | 24673073 | 46.2 | 89.8 | 23804580 | 96.48 | 18148611 | 76.24 |
| Biofilm-III | 34794660 | 45.06 | 93.1 | 24196206 | 69.54 | 18490740 | 76.42 |
| Planktonic-I | 37951770 | 44.9 | 85.1 | 35811290 | 94.36 | 26636437 | 74.38 |
| Planktonic-II | 53229419 | 43.53 | 92.4 | 52867458 | 99.32 | 35981591 | 68.06 |
| Planktonic-III | 50445352 | 44.5 | 93.8 | 49794606 | 98.71 | 43122128 | 86.6 |
| Total read count | 225769879 | 44.98 | 90.76 |  |  |  | 76.35333 |

**Table S4:** Top 50 significant (FDR<0.05 & log2FC > 1.5 ) upregulated genes in biofilm growth phase of *C. glabrata*.

| **Protein names** | **Gene names** | **locus** | **log2(FC)** | **p-value** | **q-value** |
| --- | --- | --- | --- | --- | --- |
| Phosphoenolpyruvate carboxykinase | PCK1 CAGL0H06633g | H:659325-660960 | 8.53726 | 5.00E-05 | 0.000133467 |
| Acetyl-coenzyme A synthetase 1 | ACS1 CAGL0L00649g | L:61770-83910 | 7.67589 | 5.00E-05 | 0.000133467 |
| Malate synthase | CAGL0L03982g | L:466896-468561 | 6.31816 | 5.00E-05 | 0.000133467 |
| Citrate synthase | CAGL0B03663g | B:367613-369005 | 5.19792 | 5.00E-05 | 0.000133467 |
| Isocitrate lyase (ICL) | ICL1 CAGL0J03058g | J:292525-294418 | 5.19782 | 5.00E-05 | 0.000133467 |
| Isocitrate dehydrogenase [NADP] | CAGL0B04917g | B:471050-479174 | 5.02915 | 5.00E-05 | 0.000133467 |
| Sortilin | CAGL0A01870g | A:191080-193778 | 4.63554 | 5.00E-05 | 0.000133467 |
| Isocitrate lyase | CAGL0L09273g | L:1006159-1007875 | 4.49859 | 5.00E-05 | 0.000133467 |
| Protein PNS1 | PNS1 CAGL0L04378g | L:510906-512580 | 4.46402 | 5.00E-05 | 0.000133467 |
| S-methyl-5’-thioadenosine phosphorylase | MEU1 CAGL0D03190g | D:330836-338579 | 4.23224 | 0.01585 | 0.0304742 |
| Citrate synthase | CAGL0L09086g | L:988138-989581 | 3.67056 | 5.00E-05 | 0.000133467 |
| GTP:AMP phosphotransferase, | ADK2 CAGL0L11902g | L:1278343-1279099 | 3.55936 | 5.00E-05 | 0.000133467 |
| Trehalase | CAGL0C04323g | C:419262-421515 | 3.3471 | 5.00E-05 | 0.000133467 |
| Outer spore wall protein 5 | CAGL0I00726g | I:52541-52931 | 3.15924 | 5.00E-05 | 0.000133467 |
| Autophagy-related protein 32 | ATG32 CAGL0H06545g | H:643446-644925 | 3.11872 | 5.00E-05 | 0.000133467 |
| Acyl-coenzyme A oxidase | POX1 CAGL0A03740g | A:377254-388401 | 3.09881 | 0.0259 | 0.0476413 |
| 60S ribosomal protein L2 | RPL2 CAGL0J02354g | J:227225-234772 | 3.08906 | 0.02295 | 0.0426345 |
| Transcriptional activator | HAP5 CAGL0K09900g | K:959121-972091 | 2.65428 | 0.00775 | 0.0157768 |
| Serine/threonine-protein phosphatase | CAGL0H04851g | H:462565-464314 | 2.50726 | 5.00E-05 | 0.000133467 |
| Mitochondrial pyruvate carrier | CAGL0F08085g | F:796792-801886 | 2.47045 | 5.00E-05 | 0.000133467 |
| Protein ZIP2 | ZIP2 CAGL0H07645g | H:747507-749538 | 2.45461 | 5.00E-05 | 0.000133467 |
| GTPase-activating protein GYP7 | GYP7 CAGL0C05489g | C:516402-530836 | 2.45444 | 5.00E-05 | 0.000133467 |
| 3-isopropylmalate dehydrogenase | LEU2 CAGL0H03795g | H:356534-357632 | 2.39002 | 5.00E-05 | 0.000133467 |
| NAD-specific glutamate dehydrogenase | GDH2 CAGL0G05698g | G:541467-544755 | 2.34527 | 5.00E-05 | 0.000133467 |
| 3-isopropylmalate dehydratase | CAGL0A00363g | A:33994-36349 | 2.29784 | 5.00E-05 | 0.000133467 |
| Ammonium transporter | CAGL0I10747g | I:1058859-1071757 | 2.26779 | 5.00E-05 | 0.000133467 |
| pH-response regulator protein palH/RIM21 | RIM21 CAGL0M03663g | M:411532-413152 | 2.19669 | 5.00E-05 | 0.000133467 |
| Lysophospholipase | CAGL0H03575g | H:331302-332847 | 2.10024 | 5.00E-05 | 0.000133467 |
| 3-hydroxyisobutyryl-CoA hydrolase | CAGL0F05071g | F:514360-515221 | 2.09139 | 5.00E-05 | 0.000133467 |
| Isocitrate dehydrogenase [NADP] | CAGL0H03663g | H:337532-338789 | 2.0779 | 5.00E-05 | 0.000133467 |
| ATP-binding cassette transporter CGR1 | PDH1 CGR1 CAGL0F02717g | F:71960-346856 | 1.9741 | 0.0196 | 0.0368654 |
| FAS1 domain-containing protein CAGL0M08734g | CAGL0M08734g | M:869006-869768 | 1.96535 | 5.00E-05 | 0.000133467 |
| Citrate synthase | CAGL0H03993g | H:372154-373573 | 1.93233 | 5.00E-05 | 0.000133467 |
| Phosphotransferase | CAGL0B00726g | B:63902-65153 | 1.8897 | 5.00E-05 | 0.000133467 |
| Autophagy-related protein 2 | ATG2 CAGL0J07634g | J:743066-747830 | 1.88212 | 5.00E-05 | 0.000133467 |
| Phosphatidylglycerol/phosphatidylinositol transfer protein (PG/PI-TP) | NPC2 CAGL0K01353g | K:121966-122524 | 1.87665 | 5.00E-05 | 0.000133467 |
| pH-response regulator protein palI/RIM9 | RIM9 CAGL0F06545g | F:645358-646099 | 1.86371 | 5.00E-05 | 0.000133467 |
| Acetyl-CoA hydrolase | ACH1 CAGL0J04268g | J:389833-402280 | 1.8599 | 0.0067 | 0.0137471 |
| Autophagy-related protein 11 | ATG11 CAGL0H08558g | H:832641-840908 | 1.84666 | 5.00E-05 | 0.000133467 |
| Putative redox protein FMP46, mitochondrial | FMP46 CAGL0M05951g | M:626329-626719 | 1.83394 | 5.00E-05 | 0.000133467 |
| Putative lipase ATG15 | ATG15 CAGL0J03542g | J:338755-340270 | 1.82857 | 5.00E-05 | 0.000133467 |
| Autophagy-related protein 33 | ATG33 CAGL0B02860g | B:278006-278657 | 1.81928 | 5.00E-05 | 0.000133467 |
| Myosin-5 | MYO5 CAGL0K03487g | K:317196-326546 | 1.80794 | 5.00E-05 | 0.000133467 |
| Enoyl-[acyl-carrier-protein] reductase | ETR1 CAGL0B04323g | B:422686-423979 | 1.71231 | 5.00E-05 | 0.000133467 |
| Endopolyphosphatase | PPN1 CAGL0K06237g | K:605407-607399 | 1.65404 | 5.00E-05 | 0.000133467 |
| Mitochondrial import inner membrane translocase subunit TIM22 | TIM22 CAGL0G05654g | G:534042-539098 | 1.64878 | 0.01475 | 0.0286302 |
| Peroxisomal biogenesis factor 6 | PEX6 CAGL0D02574g | D:263062-266116 | 1.64146 | 5.00E-05 | 0.000133467 |
| Aconitate hydratase | ACO1 CAGL0D06424g | D:602495-608071 | 1.60166 | 5.00E-05 | 0.000133467 |
| Ditrans,polycis-polyprenyl diphosphate synthase | CAGL0K03355g | K:305790-306819 | 1.58379 | 5.00E-05 | 0.000133467 |
| Autophagy-related protein 9 | ATG9 CAGL0I03652g | I:310646-313433 | 1.57659 | 5.00E-05 | 0.000133467 |
