# Supplementary material for: Decoding the role of oxidative stress resistance and alternative carbon substrate assimilation in the mature biofilm growth mode of Candida glabrata
Source: BMC Microbiol. 2024 Apr 20;24:128. doi: 10.1186/s12866-024-03274-9 (PMC11031924; doi:10.1186/s12866-024-03274-9)
Supplement: Supplementary file 2 — Supplementary Material 2 [file 12866_2024_3274_MOESM2_ESM.docx]

**Figure S1:** **RNA integrity of the biofilm growth phase of *C. glabrata* ( I, II & III)**: Quality of RNA isolated from three replicates of the biofilm growth phase (biofilm -I, II & III) analysed with bioanalyzer (Agilent 2100). RIN assigned to each replicate is demonstrated in figure a, b, and c respectively. X−axis represents banding mobility traces of different sizes of RNA molecules and Y-axis depicts the corresponding fluorescence.

**Figure S2:** **RNA integrity of *C. glabrata* planktonic growth phase ( I, II &III)**: Quality of isolated RNA from three replicate of the planktonic growth phase (planktonic-I, II & III) analysed with bioanalyzer (Agilent 2100). RIN assigned to each replicate is demonstrated in figure a, b, and c respectively. X−axis represents banding mobility traces of different sizes of RNA molecules and Y-axis depicts the corresponding fluorescence.

**Figure S3:** **Scatter plot of DEGs under biofilm and planktonic growth phase of *C. glabrata* (biofilm vs. planktonic)**: Scatter plots generated using the CummeRbund package illustrates the gene expression data sets between the biofilm and planktonic growth phase. X and Y-axis represents the expression of genes (FPKM values) in planktonic and biofilm growth phase of *C. glabrata* respectively, where dots represent the expressed genes.

**Figure S4 : Volcano plot matrix:** Volcano plot constructed with DEGs of replicate within the same (biofilm vs. biofilm & planktonic vs. planktonic) and different (biofilm vs. planktonic) experimental conditions.

**Figure S5:** **Visualization of enriched gene ontology (GO) terms**: The histogram and bar charts of gene ontology terms with percentage of up-regulated genes involved in biofilm formation of *C. glabrata*.

**Figure S6:** **PCR amplification of the 5’ and 3’UTR of Cg*PCK1* gene from wild-type genomic DNA of *C. glabrata* (NCCPF- 100037) and construction of a gene deletion cassette with the *Nat1* gene**: (a) The lanes 1-8 &10 of agarose gel (1.5%) show the PCR amplified product of a 641bp from 5’ untranslated regions (UTR) of Cg*PCK1* gene from wild-type genomic DNA of *C. glabrata*. Lanes 9-10 & 12-13 represent a PCR amplified product of a size a 654bp from the 3’UTR, where M is the 100bp marker (b) Show the fusion products (FP) of amplified 5’& 3’UTR of Cg*PCK1* gene with one-half each from the ends of *Nat1* gene using fusion PCR. The amplified 5’UTR + one half of *Nat1* gene resulted into a fusion product 1(FP1) of size 1403bp shown in lane1 and lane 2 show the FP2 of size 1519bp produced from amplified 3’UTR + second half of *Nat1* gene, where M is the molecular marker of size 1kb.

**Figure S7:** **PCR amplification of the 5’ and 3’UTR of Cg*PEP1* gene from wild-type genomic DNA of *C. glabrata* (NCCPF- 100037) and construction of a gene deletion cassette with the *Nat1* gene**: (a) Lanes 1-9 of the agarose gel (1.5%) show the amplified PCR amplified product of a size 615bp from 5’UTR of Cg*PEP1* gene from wild-type genomic DNA of *C. glabrata*. The lanes 10-16 show PCR amplified product of a size 625bp from the 3’UTR of Cg*PEP*1, where M is the 100bp marker (b) Show the fusion products (FP) of amplified 5’&3’UTR of Cg*PEP1* gene with one-half each from ends of *Nat1* gene using fusion PCR. The amplified 5’UTR + one half of *Nat1* gene resulted into a fusion product 1 (FP1) of size 1377bp in lane-1 and amplified 3’UTR + second half of *Nat1* gene resulted into a FP2 of size 1490bp in lane-2, where M is the molecular marker of size 1kb.

**Figure S8:** **Cg*PCK1* knockout verification in Cg*pck1***∆ **mutant (transformants) and wild type *C. glabrata* (NCCPF-100037)**: Lanes 2,4,6,7 & 8-10 represent the amplified PCR products of size 573bp from full *Nat1* gene cassette inserted in place of Cg*PCK1* gene in Cg*pck1*∆ mutant strain. No amplification was observed from genomic DNA of wild type *C. glabrata* (NCCPF-100037) in lane 13, lanes 1 & 14 show the molecular marker M of size 100bp. The lane-15 show the PCR amplified product of a size 334bp using internal primer for gene Cg*PCK1* with genomic DNA from wild type *C. glabrata* (NCCPF-100037) and taken as a positive control whereas, lane-16 is a negative control with no template DNA showed no amplification. Lanes 18-19 & 21-27 showed no PCR amplification using internal primer for gene *CgPCK1* with genomic DNA isolated from the Cg*pck1*∆ mutant (transformants), conversely lane 4 showed PCR amplified product of a size 334bp with Cg*pck1*∆ mutant DNA.

**Figure S9:** **Gene knockout verification of Cg*PEP1* gene in Cg*pep1***∆ **mutant (transformants) and wild type *C. glabrata* (NCCPF-100037)**: (a) Lanes 2-11 represent the amplified PCR products of size 573bp from full *Nat1* gene cassette inserted in place of Cg*PEP1* gene in Cg*pep1*∆ mutant strain. No amplification was observed from genomic DNA of wild type *C. glabrata* (NCCPF-100037) in lane 13, lanes 1 shows the molecular marker M of size 100bp (b) The lane 2 show the PCR amplified product of a size 302bp using internal primer for gene Cg*PEP1* with genomic DNA from wild type *C. glabrata* (NCCPF-100037) and taken as a positive control. Lanes 4-17 showed no PCR amplification using internal primer for gene Cg*PEP1* with genomic DNA isolated from the Cg*pep1*∆ mutant (transformants), conversely lane 3 showed PCR amplified product of a size 302bp with Cg*pep1*∆ mutant DNA.
